# Role and effectiveness of complex and supervised rehabilitation on overall and hand function in systemic sclerosis patients—one-year follow-up study

**DOI:** 10.1038/s41598-021-94549-y

**Published:** 2021-07-26

**Authors:** Michał Waszczykowski, Bożena Dziankowska-Bartkowiak, Michał Podgórski, Jarosław Fabiś, Arleta Waszczykowska

**Affiliations:** 1 Department of Arthroscopy, Minimally Invasive Surgery and Sports Traumatology, Chair of Orthopaedics, Traumatology and Rehabilitation, Medical University of Lodz, Kosciuszki 4, 90-419 Lodz, Poland; 2 Department of Dermatology and Venereology, Medical University of Lodz, Plac Hallera 1, 90-647, Lodz, Poland; 3 Department of Diagnostic Imaging, Polish Mother’s Memorial Hospital Research Institute, Rzgowska 281/289, 93-338 Lodz, Poland; 4 Department of Ophthalmology and Vision Rehabilitation, Medical University of Lodz, Zeromskiego 113, 90-549 Lodz, Poland

**Keywords:** Bone, Muscle, Skeleton, Musculoskeletal abnormalities, Systemic sclerosis, Musculoskeletal system

## Abstract

The aim of this study was to estimate the long-term results of complex and supervised rehabilitation of the hands in systemic sclerosis (SSc) patients. Fifty-one patients were enrolled in this study: 27 patients (study group) were treated with a 4-week complex, supervised rehabilitation protocol. The control group of 24 patients was prescribed a home exercise program alone. Both groups were evaluated at baseline and after 1-, 3-, 6-, and 12-months of follow-up with the Disability of the Arm, Shoulder and Hand Questionnaire (DAHS) as the primary outcome, pain (VAS—visual analog scale), Cochin Hand Function Scale (CHFS), Health Assessment Questionnaire Disability Index (HAQ-DI), Scleroderma-HAQ (SHAQ), range of motion (d-FTP—delta finger to palm, Kapandji finger opposition test) and hand grip and pinch as the secondary outcomes. Only the study group showed significant improvements in the DASH, VAS, CHFS and SHAQ after 1, 3 and 6 months of follow-up (P = 0.0001). Additionally, moderate correlations between the DASH, CHFS and SHAQ (R = 0.7203; R = 0.6788; P = 0.0001) were found. Complex, supervised rehabilitation improves hand and overall function in SSc patients up to 6 months after the treatment but not in the long term. The regular repetition of this rehabilitation program should be recommended every 3–6 months to maintain better hand and overall function.

## Introduction

Systemic sclerosis (SSc, scleroderma) is a severe, chronic autoimmune connective tissue disease characterized by skin thickening, Raynaud’s phenomenon, visceral organ damage and musculoskeletal involvement^1^. Systemic sclerosis is divided into two basic categories based on the extent of pathological changes: limited cutaneous systemic sclerosis (lcSSc), in which the hardened skin lesions do not exceed ^1^/_3_ of the forearm length and can occur on the face, and diffuse cutaneous systemic sclerosis (dcSSc), which is characterized by generalized hardening that affects a large area of the skin. The involvement of internal organs is more prominent and aggressive in the latter and is caused by fibroproliferative alterations in the microvasculature, which lead to the excessive deposition of collagen fibers^1–4^. Progressive pathological changes in the skin, internal organs and musculoskeletal system gradually cause dysfunction and affect quality of life^5,6^.

The pathological changes in scleroderma very often affect the hands, which significantly limits patients’ dexterity and the ability to perform daily activities. The thickening of the skin and subcutaneous tissue, fibrosis of the tendons and palmar aponeurosis result in contractures of the fingers, limiting flexion in the metacarpophalangeal joints (MCP) and extension in the proximal and distal interphalangeal joints (PIP, DIP). This leads to a claw-type deformity, with metacarpophalangeal joint (MCP) extension, proximal and distal interphalangeal joint (PIP, DIP) flexion and thumb adduction and limited wrist range of motion (ROM)^5–12^. Inflammatory arthritis, tendon friction rubs (TFR), joint contractures, Raynaud’s phenomenon (RP), puffy hands, digital ulcers (DU), skin sclerosis and necrosis, calcinosis and acro-osteolysis are often coexisting manifestations in SSc patients and impair patient quality of life^13,14^.

Despite the knowledge of the significant impact of pathological changes in scleroderma on the function of the musculoskeletal system, the treatment of SSc focuses primarily on skin lesions and internal organ complications. Concurrent proper treatment of musculoskeletal system complications, especially those of the upper limbs, can lead to significant, global improvements in SSc treatment results and patient quality of life^15,16^.

Proper, well-organized rehabilitation of hand function combined with other therapies may play an important role in treating the musculoskeletal complications of scleroderma. There are only a few studies in the literature showing the influence of specific rehabilitation techniques on improvements in hand function in scleroderma patients^14–22^.

Reliable data on the long-term outcomes of complex, standardized, supervised hand rehabilitation in patients with SSc and the effect of time on the gradual loss of hand function are lacking.

The primary aim of this study was to determine the long-term effects of complex, standardized, supervised rehabilitation involving whirlpool massage combined with active exercises of the upper limb, massage of the soft tissues of the upper limb and manipulation of the hand joints on reducing pain and improving hand function in patients with systemic sclerosis. To assess the pain, function and muscular strength of the hand and global disability in SSc patients, the visual analog scale (VAS pain); Disability of the Arm, Shoulder and Hand Questionnaire (DASH)^23–27^; Cochin Hand Function Scale (CHFS)^25–27^; Health Assessment Questionnaire Disability Index (HAQ-DI); Scleroderma-HAQ (SHAQ)^28–31^; delta finger-to-palm (d-FTP) test^32^; Kapandji finger opposition test; and hand grip and pinch measurements were used^33,34^.

The secondary aim was to estimate how long the positive effect of the applied rehabilitation on hand function could be maintained in patients with SSc and how often these patients should undergo this type of treatment.

## Results

Fifty one patients were enrolled in this study. The baseline characteristics of the patients in both groups are presented in Table 1. There were no statistically significant differences between the two groups in terms of age and sex (Table 1). All the patients in the study group finished the entire one-month physiotherapy program. Two patients in the study group and one in the control group did not reach the last follow-up point (after 12 months).

**Table 1:** Baseline clinical and demographical characteristics of the study and control groups.

Clinical characteristics	Study group (n = 27)	Control group (n = 24)
Age: mean, range (± SD)	54.5, 35–77 (10.1)	55.2, 38–67 (9.2)
Women/men (%)	25/2 (92.6%/7.4%)	22/2 (91.7%/8.3%)
Skin score (mRSS): mean (± SD)	12.9 (7.4)	13.2 (6.2)
**Type of SSc**		
lcSSc	18	17
dcSSc	9	7
Duration of the SSc (*), mean, range (± SD)	11, 3–22 (7.5)	12.3, 4–18 (6.7)
Gastrointestinal manifestation	16 (59.3%)	13 (54.2%)
Pulmonary fibrosis	11 (40.6%)	8 (33.2%)
Cardiac involvement	17 (63%)	12 (50%)
Renal abnormalitis	4 (14.7%)	1 (4.2%)
Hematological involvement	6 (22.1%)	5 (20.7%)

The outcomes are shown as a numbers and percentages. Values are the means, ranges and ± SDs. Cardiac involvement: cardiac myositis, coronary artery disease, arrhythmia. Pulmonary fibrosis: Only one patient (3.6%) in the study group had pulmonary arterial hypertension (PAH); none (0%) in the control group had PAH.

*SSc* systemic sclerosis, *lcSSc* limited cutaneous systemic sclerosis, *dcSSc* diffuse cutaneous systemic sclerosis, *mRSS* modified Rodnan skin score.

The results for the primary outcome of DASH and the secondary outcomes of the VAS, CHFS, HAQ-DI, SHAQ, CHFS, FTP, dFTP, Kapandji score and hand muscle strength (hand grip and pinch) are shown in Table 2.

**Table 2:** Characteristics of measurements changes of SSc patients at baseline (Day 0) and 1, 3, 6 and 12-month-follow-up.

Parameter			Day 0		P*	Month 1		P*	Month 3		P*	Month 6		P*	Month 12		P*	P†
			**SSc**	**Control**		**SSc**	**Control**		**SSc**	**Control**		**SSc**	**Control**		**SSc**	**Control**		
**DASH**			49.4 (11.8)	52.3 (10.7)	0.5197	33.1 (9.6)	50.3 (10.4)	0.0002	36.3 (10.4)	53.1 (10.7)	0.0006	42.6 (11.5)	53.4 (10.6)	0.0209	48.3 (12.7)	53.8 (10.9)	0.2500	0.0001
**VAS**			5.3 (1.5)	5.3 (1.1)	1.0000	2.9 (1.1)	4.7 (1.1)	0.0003	3.3 (1.2)	5.0 (1.0)	0.0006	4.1 (1.2)	5.0 (1.0)	0.0582	5.1 (1.7)	5.4 (0.9)	0.3906	0.0001
**CHFS**			42.3 (11.0)	41.2 (12.1)	0.6756	27.9 (7.5)	38.6 (12.0)	0.0026	30.4 (8.2)	41.7 (11.9)	0.0031	36.1 (10.2)	41.5 (11.4)	0.2189	40.6 (10.9)	42.3 (11.9)	0.7573	0.0001
**HAQ-DI**			1.3 (0.5)	1.4 (0.6)	0.6407	0.9 (0.5)	1.4 (0.6)	0.0323	1.1 (0.7)	1.4 (0.6)	0.0515	1.1 (0.6)	1.5 (0.6)	0.1000	1.3 (0.6)	1.5 (0.5)	0.4499	0.0001
**SHAQ**	**ALL**		1.3 (0.5)	1.3 (0.5)	0.6874	0.9 (0.4)	1.3 (0.5)	0.0394	0.9 (0.4)	1.4 (0.5)	0.0205	1.1 (0.5)	1.4 (0.6)	0.1437	1.3 (0.5)	1.4 (0.5)	0.5827	0.0001
	**RP**		1.6 (0.6)	1.3 (0.5)	0.1263	1.1 (0.5)	1.1 (0.5)	0.6639	1.2 (0.5)	1.3 (0.5)	0.4991	1.4 (0.6)	1.4 (0.6)	0.8943	1.6 (0.6)	1.4 (0.5)	0.2644	0.0001
	**DU**		1.7 (0.6)	1.4 (0.5)	0.1615	1.0 (0.5)	1.3 (0.5)	0.1924	1.2 (0.6)	1.4 (0.5)	0.1924	1.5 (0.6)	1.5 (0.4)	0.8811	1.6 (0.6)	1.5 (0.5)	0.7835	0.0001
**dFTP dom**			5.3 (1.7)	5.6 (1.1)	0.4304	6.0 (1.7)	5.8 (1.1)	0.7233	5.9 (1.6)	5.7 (1.0)	0.7476	5.5 (1.7)	5.6 (1.0)	0.8161	5.3 (1.8)	5.6 (1.0)	0.6065	0.0001
**dFTP ndom**			5.3 (1.6)	5.8 (0.8)	0.1869	6.0 (1.5)	6.0 (0.8)	0.7233	5.9 (1.6)	5.9 (0.9)	0.8469	5.6 (1.6)	5.7 (0.9)	0.6182	5.4 (1.6)	5.6 (0.8)	0.3628	0.0001
**Kapandi**		**dom**	5.9 (1.4)	5.7 (1.0)	0.6179	7.0 (1.7)	6.7 (1.0)	0.3675	6.7 (1.4)	6.1 (1.3)	0.2466	6.1 (1.5)	5.7 (1.2)	0.4156	5.9 (1.6)	5.4 (1.2)	0.2949	0.0001
		**ndom**	5.9 (1.1)	5.5 (1.4)	0.4689	7.0 (1.4)	5.8 (1.3)	0.0253	6.6 (1.3)	5.5 (1.2)	0.0223	6.1 (1.2)	5.3 (1.3)	0.0628	5.8 (1.2)	5.2 (1.4)	0.1919	0.0001
**Hand grip**		**dom**	7.7 (2.8)	8.4 (1.9)	0.7721	8.5 (3.1)	8.6 (1.9)	0.7354	8.2 (3.0)	8.3 (1.6)	0.7476	7.7 (2.9)	8.1 (1.6)	0.9074	7.5 (2.9)	7.9 (1.6)	0.9726	0.0001
		**ndom**	8.1 (2.6)	7.7 (1.7)	0.7844	8.7 (2.8)	7.7 (1.7)	0.3590	8.5 (2.7)	7.5 (1.6)	0.3184	8.1 (2.6)	7.4 (1.6)	0.4648	7.9 (2.5)	7.2 (1.5)	0.4499	0.0001
**Hand pinch**		**dom**	3.6 (1.1)	3.7 (1.1)	1.0000	4.1 (1.1)	3.7 (1.2)	0.2738	4.0 (1.1)	3.6 (1.1)	0.3030	3.7 (1.1)	3.5 (1.1)	0.3876	3.6 (1.1)	3.4 (1.1)	0.6092	0.0001
		**ndom**	4.0 (1.0)	3.4 (1.0)	0.0974	4.5 (1.1)	3.5 (1.0)	0.0115	4.4 (1.1)	3.4 (1.0)	0.0126	4.1 (1.0)	3.3 (1.0)	0.0191	4.0 (1.0)	3.3 (1.0)	0.0503	0.0001

Scores of performed tests reported as a mean and ± SD in ().

*DASH* Disability of the Arm, Shoulder and Hand Questionnaire, *VAS* visual analog scale, *CHFS* The Cochin Hand Function Scale, *HAQ-DI* Health Assessment Questionnaire Disability Index, *S-HAQ* Scleroderma-HAQ, *RP* Raynaud’s Phenomenon, *DU* digital ulcers, *dFTP* Delta finger-to-palm, *dom* dominant hand, *ndom* nondominant hand, *Kapandji* The Kapandji finger opposition test; P*—p-value for the Manny-Whitney U test indicating significant results according to the Bonferroni correction (p < 0.0033), P†—p-value for the F-Omnibus test of the Friedman ANOVA, only for the study group.

In the study group, at the end of the rehabilitation program (1-month follow-up), there were statistically significant improvements in all measured parameters and scales (p = 0,0001, Table 2). The post hoc analysis showed that the significant improvements in all assessed factors were sustained after the 3-month follow-up except global disability, as measured by the HAQ-DI (Table 2). After the 6-month follow-up, the improvements in hand disability (DASH, CHFS), pain (VAS) and global disability according to the SHAQ scale remained significant (Table 2). However, after the 12-month follow-up, there were no statistically significant differences compared to the baseline results in any measured parameters (Table 2).

In the study group, we found moderate correlations between the pain assessment scale (VAS) and hand disability measurements (DASH, CHFS) (R = 0.6512; R = 0.6250, respectively; P = 0.0001; Table 3). The VAS scores were also positively correlated with the global disability assessment (SHAQ) (R = 0.6560; P = 0.0001; Table 3). Our analysis also indicated moderate positive correlations between hand function assessments (DASH, CHFS) and the global disability evaluation (SHAQ) (R = 0.7203 and R = 0.6788, respectively; P = 0.0001; Table 3). Table 3 presents all the correlations of the analyzed parameters. To avoid redundancy, data were pooled from all time points and, if appropriate, scores for the dominant hand were included.

**Table 3:** Correlations of studied parameters.

	**DASH**	**VAS**	**CHFS**	**HAQ-DI**	**SHAQ ALL**	**dFTP**	**Kapandji**	**Hand grip**	**Hand pinch**
DASH	–	R = 0.6512 P = 0.0001	R = 0.8674 P = 0.0001	R = 0.5397 P = 0.0001	R = 0.7203 P = 0.0001	R^2^ = 0.533 P = 0.0001	R = 0.3275 P = 0.0001	R = 0.3499 P = 0.0001	R = 0.5621 P = 0.0001
VAS	R = 0.6512 P = 0.0001	–	R = 0.6250 P = 0.0001	R = 0.4388 P = 0.0001	R = 0.6560 P = 0.0001	R^2^ = 0.339 P = 0.0001	R = 0.3316 P = 0.0001	R = 0.1841 P = 0.0001	R = 0.3138 P = 0.0001
CHFS	R = 0.8674 P = 0.0001	R = 0.6250 P = 0.0001	–	R = 0.5398 P = 0.0001	R = 0.6788 P = 0.0001	R^2^ = 0.474 P = 0.0001	R = 0.2391 P = 0.0001	R = 0.4168 P = 0.0001	R = 0.5439 P = 0.0001
HAQ-DI	R = 0.5397 P = 0.0001	R = 0.4388 P = 0.0001	R = 0.5398 P = 0.0001	–	R = 0.7011 P = 0.0001	R^2^ = 0.420 P = 0.0001	R = 0.2019 P = 0.0001	R = 0.3701 P = 0.0001	R = 0.3756 P = 0.0001
SHAQ ALL	R = 0.7203 P = 0.0001	R = 0.6560 P = 0.0001	R = 0.6788 P = 0.0001	R = 0.7011 P = 0.0001	–	R^2^ = 0.570 P = 0.0001	R = 0.3589 P = 0.0001	R = 0.3974 P = 0.0001	R = 0.5281 P = 0.0001
dFTP	R^2^ = 0.533 P = 0.0001	R^2^ = 0.339 P = 0.0001	R^2^ = 0.474 P = 0.0001	R^2^ = 0.420 P = 0.0001	R^2^ = 0.570 P = 0.0001	–	R^2^ = 0.153 P = 0.0001	R^2^ = 0.481 P = 0.0001	R^2^ = 0.437 P = 0.0001
Kapandji	R = 0.3275 P = 0.0001	R = 0.3316 P = 0.0001	R = 0.2391 P = 0.0001	R = 0.2019 P = 0.0001	R = 0.3589 P = 0.0001	R^2^ = 0.153 P = 0.0001	–	R = 0.0635 P = 0.0036	R = 0.2936 P = 0.0001
Hand grip	R = 0.3499 P = 0.0001	R = 0.1841 P = 0.0001	R = 0.4168 P = 0.0001	R = 0.3701 P = 0.0001	R = 0.3974 P = 0.0001	R^2^ = 0.481 P = 0.0001	R = 0.0635 P = 0.0036	–	R = 0.5716 P = 0.0001
Hand pinch	R = 0.5621 P = 0.0001	R = 0.3138 P = 0.0001	R = 0.5439 P = 0.0001	R = 0.3756 P = 0.0001	R = 0.5281 P = 0.0001	R^2^ = 0.437 P = 0.0001	R = 0.2936 P = 0.0001	R = 0.5716 P = 0.0001	–

*VAS* visual analog scale, *DASH* Disability of the Arm, Shoulder and Hand Questionnaire, *CHFS* The Cochin Hand Function Scale, *HAQ-DI* Health Assessment Questionnaire Disability Index, *S-HAQ* Scleroderma-HAQ, *dFTP* Delta finger-to-palm (for dominant hand), *Kapandji* The Kapandji finger opposition test (for dominant hand); Hand grip and pinch for dominant hand. According to Bonferroni correction only Kapandji and hand grip scores did not correlate significantly. Correlations higher than 0,5 (R > 0,5) have been colored.

The control group showed slight improvement with regard to hand function (DASH, CHFS), pain (VAS) and range of motion (Kapandji score) only after the 1-month follow-up, but the improvements were not significant (Table 2).

There were no complications or adverse events related to the combined treatment.

## Discussion

Pathological changes in the musculoskeletal system are an almost universal element of SSc. Edema and pain in the hand joints, deformity, and limitations on the ROM in interphalangeal and metacarpophalangeal joints occur in nearly 80% of patients with SSc^35^. These changes cause a marked impairment of upper limb mobility in these patients, significantly limiting their ability to perform everyday activities and reducing their quality of life. Therefore, it seems that the rehabilitation of the musculoskeletal complications in these patients should be an important and permanent component of the overall treatment of SSc patients^14,35,36^. This treatment should be both personalized and adapted to the stage and phase of the disease and accompanying changes in other organs and systems^38^. According to data from a recent multicenter study in a large group of patients with SSc (n = 1627), approximately 23% of them had received rehabilitation treatment (PT/OT) in the 3 months preceding the study^35^. In 59% of all patients, the main indication for this treatment was pain in and dysfunction of the hand^35^.

**Figure 1:**
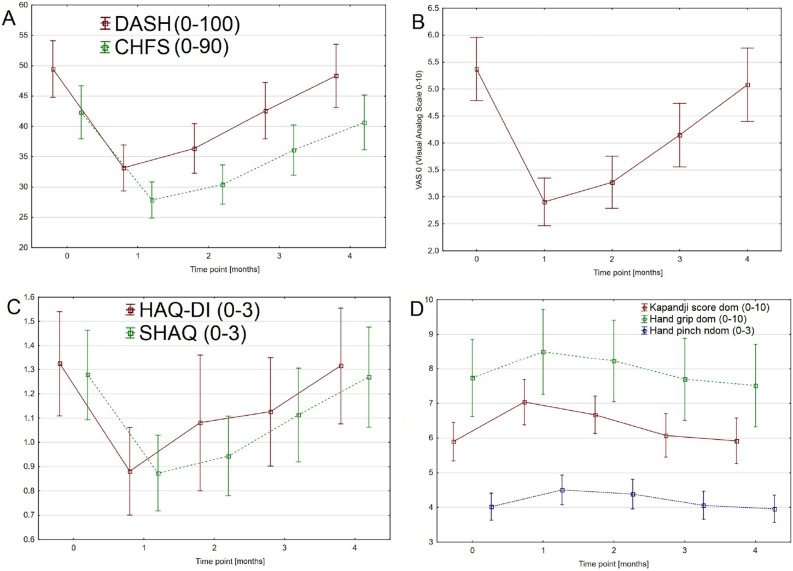
Changes of the measured parameters in study group during the time of follow-up: DASH and CHFS (**A**); VAS (**B**); HAQ-DI and SHAQ (**C**); Kapandji score, Hand grip and pinch (**D**). Time points: 0—baseline; 1—one-month-follow-up; 2—three-month-follow-up; 3—six-month-follow-up; 4—one-year-follow-up.

The results of our study indicate that the combined, standardized, supervised rehabilitation proposed here has a significant effect on improving hand function and reducing pain in SSc patients (Table 2, Fig. 1). Statistically significant improvements in hand function were achieved both immediately after the completion of the rehabilitation program and after 3 and 6 months of follow-up. However, the further assessment of hand function after the one-year follow-up indicated that between 6 and 12 months after the rehabilitation treatment, gradual reductions in hand function and the recurrence of pain may develop (Fig. 1). However, in the control group, the improvements in pain and hand function only lasted until the one-month follow-up.

To the best of our knowledge, this is the first study to show the results of a combined, standardized, supervised rehabilitation program in patients with SSc with symptoms of upper limb dysfunction; this program consisted of whirlpool massage of the upper limbs combined with active exercises of the hand and elbow joint, manual massage of the soft tissues of the hand and forearm and passive manipulation of the hand joints and wrists. The combination of these techniques was aimed at influencing all possible adverse aspects and components of hand dysfunction in SSc patients. The therapeutic whirlpool bath (WB) has a relaxing effect on soft tissues, reducing muscle tension and contraction, improving the local blood supply and reducing pain^37,38^. The therapeutic whirlpool bath created more effective conditions for the performance of the active exercises of the hand and stretching exercises^37,39^. Manual soft tissue massage influences the local blood circulation of the skin and muscles, increases the temperature of the skin by approximately 1.5 °C, causes mechanical movement of the blood in the blood vessels and lymphatic vessels, accelerates the transport of oxygen and nutrients to the massaged tissues, promotes the removal of unnecessary products of metabolism, activates a significant portion of the capillaries in the muscles that are not used at rest, and reduces muscle tension^16,40^. It increases the ability of the muscles to work, the flexibility and endurance of the ligaments and the mobility of the joint^42^. Passive and active joint mobilization and exercises improve the range of motion of the hand joints and hand function in SSc patients^15–21^.

There are only a few publications in the available literature that discuss the results of supervised hand rehabilitation programs in SSc patients^15–21,35,41^. Maddali Bongi et al., in their randomized control trial (RCT), showed that a complex rehabilitation program (manual massage, joint mobilization and daily at-home exercises) conducted twice a week for 9 weeks resulted in a statistically significant improvement in hand function compared to daily at-home exercises alone. This improvement was also noted 9 weeks after the completion of rehabilitation, according to the HAQ, Cochin scale and the Hand Mobility in Scleroderma (HAMIS) test^17^. In turn, Horvath and colleagues showed good results (after six months of follow-up) of a three-week program of intensive hand stretching exercises, ergotherapy supplemented with thermal and mud baths, whirlpool therapy and soft tissue massage compared to the control condition^20^. They obtained statistically significant improvements in hand function according to the HAQ and DASH scores after the six-month follow-up in the rehabilitation group. Murphy and colleagues in their pilot study also found statistically significant improvements in upper limb function according to the QuickDASH questionnaire and overall physical function according to the PROMIS after 8 weeks of occupational therapy treatment^21^. Their findings also supported the feasibility of the proposed 8 sessions in the complex rehabilitation protocol for early SSc patients with upper limb dysfunction^21^. In turn, Antoniolli and colleagues found in that the use of hand stretching exercises and occupational therapy, when combined with physical therapy, yielded significant improvements in the hand function of patients with SSc, according to the HAQ-DI and HAMIS test, over a 4-month follow-up period^15^. However, only Rannou et al., in their multicenter randomized control trial (RCT), reported the one-year follow-up outcomes of rehabilitation in a group of SSc patients. These results revealed no statistically significant differences between the group of SSc patients treated with the rehabilitation protocol and those who did not undergo such rehabilitation at the end of the investigation^19^. However, statistically significant improvements in hand function and reductions of pain were achieved immediately after the rehabilitation program in the rehabilitation group.

One of the most important issues in the rehabilitation of patients with chronic diseases with musculoskeletal impairments, including those with SSc, is its regularity and repeatability^16,36^. These elements are crucial for improving patients’ ability to perform activities of daily living (ADL) and preventing permanent musculoskeletal complications. Additionally, in patients with SSc with pathological changes in the musculoskeletal system, restrictions of the ROM of the hand joints, finger contractions, phalangeal ulcers, swellings and deformities, the maintenance of upper limb function is one of the most important components of systemic treatment. The proper and complex rehabilitation of these patients provides them the opportunity to perform ADLs, maintaining their family and social activities. At the same time, it seems that the second most important element in the process of the rehabilitation of these patients is its regularity. The determination of the frequency of rehabilitation should be based primarily on its effectiveness and the period of time after which the improvement disappears. Our study is probably the first to estimate how often this combined rehabilitation program should be performed to preserve the improved function of the hands in SSc patients. In this study, we attempted to answer the question of how often combined, standardized, supervised rehabilitation programs should be repeated for SSc patients. To this end, we assessed the impact of the complex rehabilitation program on hand function in patients with SSc after the end of treatment and after 3, 6 and 12 months of follow-up. The analysis of the results indicated that statistically significant improvements in hand function and reductions of pain persisted for up to 6 months after rehabilitation. After that time, the recurrence of pain and loss of hand function were observed (Fig. 1). It seems, therefore, that the regularity of the conducted rehabilitation is crucial for maintaining hand function and the ability to perform ADL in patients with SSc. Furthermore, the correlations among the outcomes in our study may indicate significant relationships between the level and severity of hand pain and hand function in these patients. These relationships in turn may have direct, positive impacts on overall satisfaction and QOL in SSc patients. The results of our study indicate that we should consider establishing a recommendation to repeat this combined, standardized, supervised rehabilitation protocol for SSc patients every 3–6 months. At the same time, it seems important to conduct further research in this field to specify the time at which the pain and loss of hand function start to recur.

Unfortunately, there are some limitations of this study. One limitation is the relatively small size of the study group. However, taking into account the rarity of this disease, the severity of its course and the possibility of regular participation in subsequent rehabilitation sessions, it may be problematic to gather more patients in one research center and conduct a full one-year follow-up analysis. Taking into account the numbers of patients in the other available publications, it seems that the sample size in our study has been optimized^15–18^. Another important limitation of our study may be the lack of randomization. The randomization and blindness of research groups in studies on patients with this type of disease and this kind of treatment are controversial and problematic^15,17^. The combined rehabilitation program introduced many variables that made randomization difficult, but it is also more beneficial for patient health and the best and most complex rehabilitation strategy for these patients. Moreover, the blindness of research groups is more difficult to achieve in these studies than in studies on the impact of pharmacological treatment (monotherapy). Withdrawal from participation in the study is a frequent phenomenon among patients who find that they have been assigned to a control group^18^. Patients in the control group were recruited from among patients who were unable to participate in the supervised rehabilitation program at the time of the study.

The results of our study show the significant role of the proposed combined program of upper limb rehabilitation in systemic sclerosis patients with hand dysfunction. The improvements in pain, global disability and hand function after physiotherapy were maintained from 3 to 6 months after program completion. The analysis of the outcomes indicated strong correlations among hand pain, hand disability and global disability in these patients. It seems highly justified to recommend the repetition of this complex rehabilitation program at least twice per year to significantly improve hand and overall function in SSc patients. Therefore, further research in this field should be conducted to precisely determine the appropriate frequency of this treatment.

## Conclusions

The results of our study show the important role of the proposed complex program of rehabilitation of the upper limbs in systemic sclerosis patients with hand dysfunction. The improvements in pain, overall function and hand function after physiotherapy were maintained from 3 to 6 months after program completion. The analysis of the outcomes indicates moderate correlations among hand pain, hand disability and global disability in these patients. It seems highly justified to recommend the repetition of this complex rehabilitation program at least twice per year to significantly improve hand and overall function in SSc patients. Therefore, further research in this field should be conducted to precisely determine the appropriate frequency of this treatment.

## Materials and methods

### Study design and study and control groups

A longitudinal two-arm interventional study was conducted in 51 patients who met the criteria for the diagnosis of systemic sclerosis (SSc) according to the ACR/EULAR 2013 Classification Criteria^42^. The other eligibility criteria were age ≥ 18 years (adult), contracture or limitation of the range of motion in at least one joint of the hand and willingness to participate in the entire rehabilitation program. Additional characteristics of the studied groups are presented in Table 1.

All patients were consecutively enrolled in this study from the outpatient clinic of the Department of Dermatology and Venerology of the University of Lodz (Poland) and were then referred to the outpatient orthopedics clinic and the physiotherapy and rehabilitation outpatient department. All the patients gave their written informed consent to participate in the study. The study was conducted in accordance with the Declaration of Helsinki. Ethics approval for the study was obtained from the Bioethics Committee of the Medical University of Lodz (approval number RNN/332/06/KB). The main exclusion criteria were a history of other autoimmune diseases, cancer, dysfunction of the upper limbs caused by past injury and participation in a similar rehabilitation protocol during the past 6 months.

**Figure 2:**
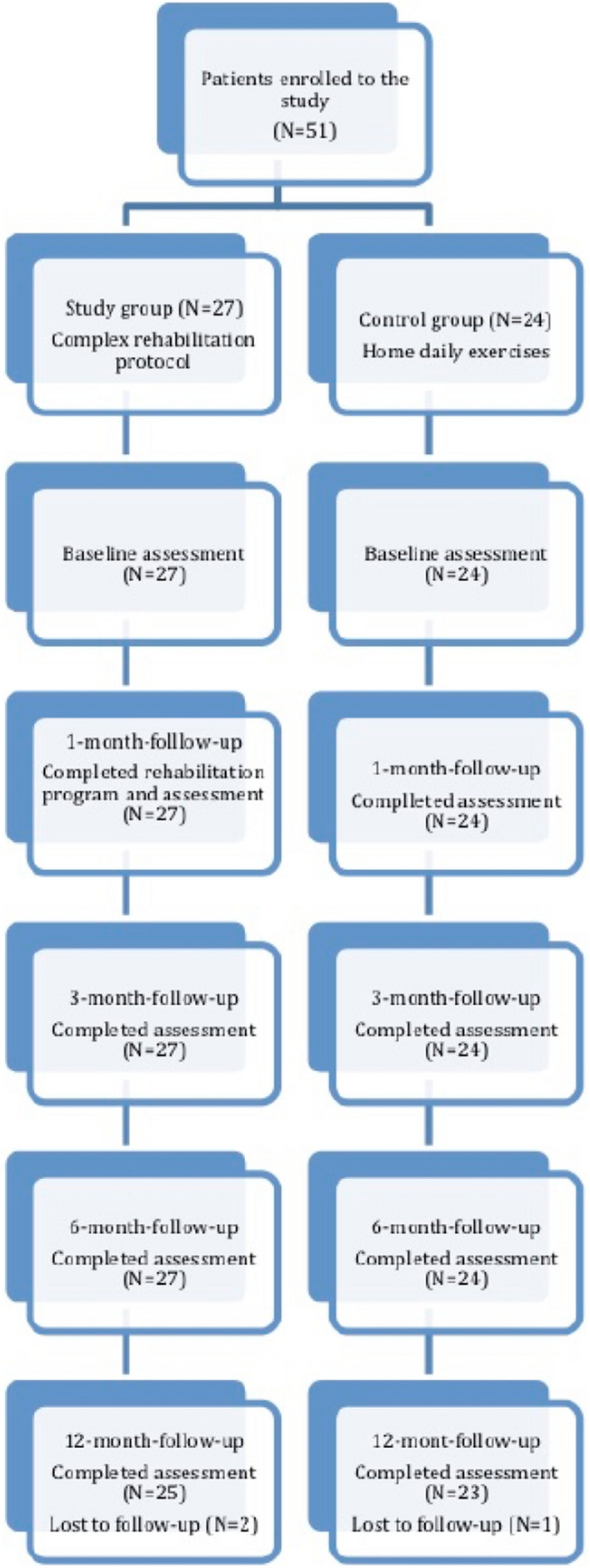
Participants flow in the study.

All patients were assigned to one of two groups: the study (n = 27) and control (n = 24) groups. The patients in the study group underwent a standardized, complex, supervised physiotherapy program. The patients in the control group followed an at-home daily exercise protocol and could not take part in the supervised rehabilitation program at the time of the study. The participant flow chart is shown in Fig. 2.

The lcSSc patients were treated with vasodilating drugs (angiotensin receptor antagonists, calcium channel blockers, or pentoxifylline) and vitamin E. The dcSSc patients were treated with immunosuppressive therapy (low doses of corticosteroids—prednisone 0.5 mg/kg bw/day, methylprednisone 8 mg) alone or in combination with cytostatic therapy (cyclophosphamide 1.5 mg/kg bw/day), vitamin E, pentoxifylline (Polfilin), mucolytic agents (acetylcysteine), omeprazole (Bioprazole), and dextran infusion.

### Clinical examination

**Figure 3:**
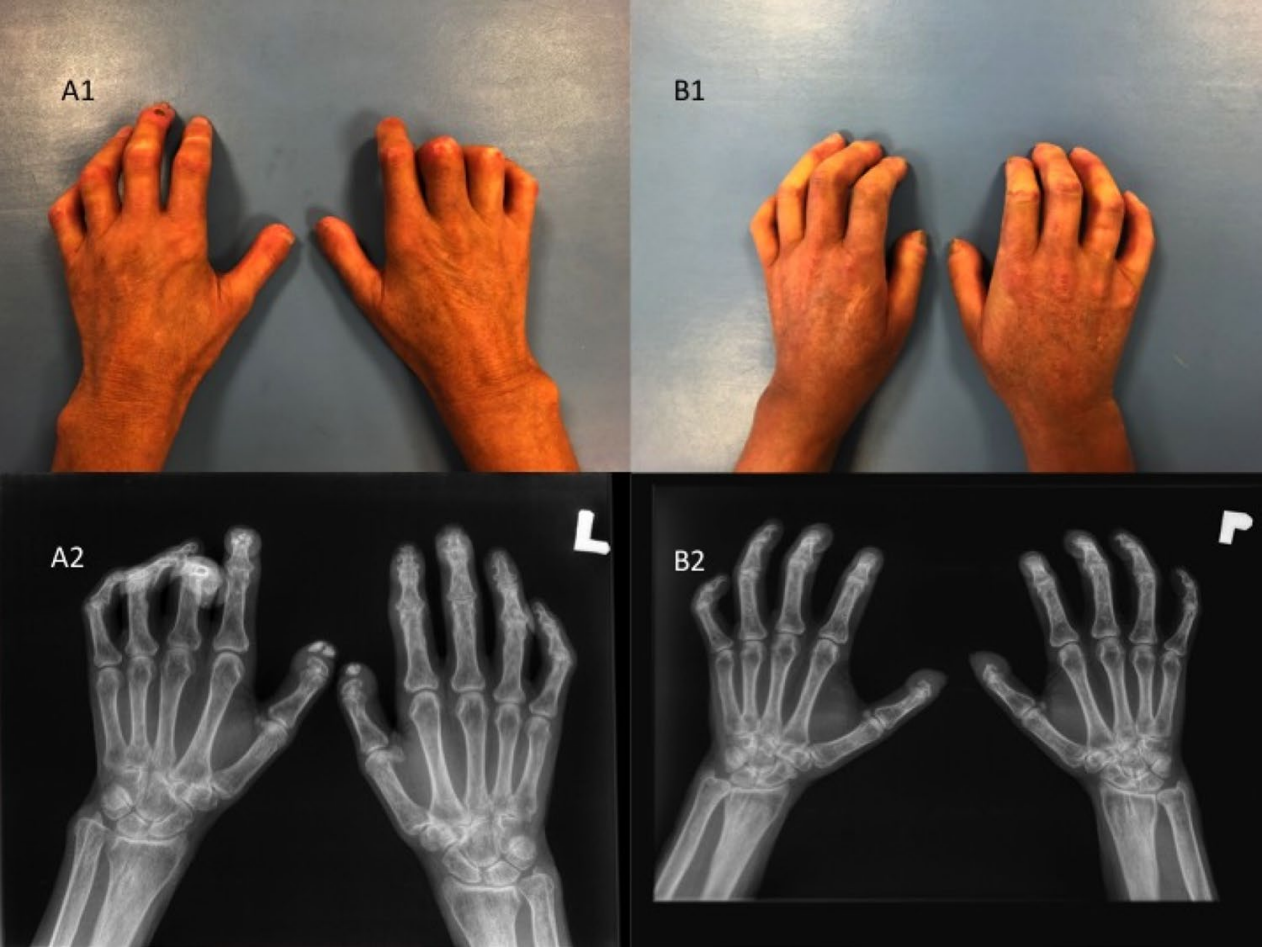
Clinical and radiological manifestations of pathological changes in the hands in SSc patients’: thickening of the skin and subcutaneous tissue (**A1**,**B1**); the palpable fibrosis of the palmar apponeurosis and flexores muscles tendons of the fingers (**A1**,**B1**); claw-type deformity of the fingers: a limited extension in proximal and distal interphalangeal joints (PIP, DIP) (**A1**,**B1**); a hyperextension in metacarpophalangeal joints (MCP) (**A1**,**B1**); the digital ulcer (DU) of distal phalanx of the 3th finger of the left hand (**A1**). Resorption of bilateral 1st, 2nd and 3rd distal phalanxes (**A2**,**B2**); joint space narrowing of MCP, PIP and DIP joints (**A2**,**B2**); errosions and juxta-articular osteopenia (**A2**,**B2**); acro-osteolysis of the distal phalanxes of the thumbs (A2) and 1st, 2nd, 3rd of the both hands **B2**); calcinosis of the first fingertips (**A2**).

**Figure 4:**
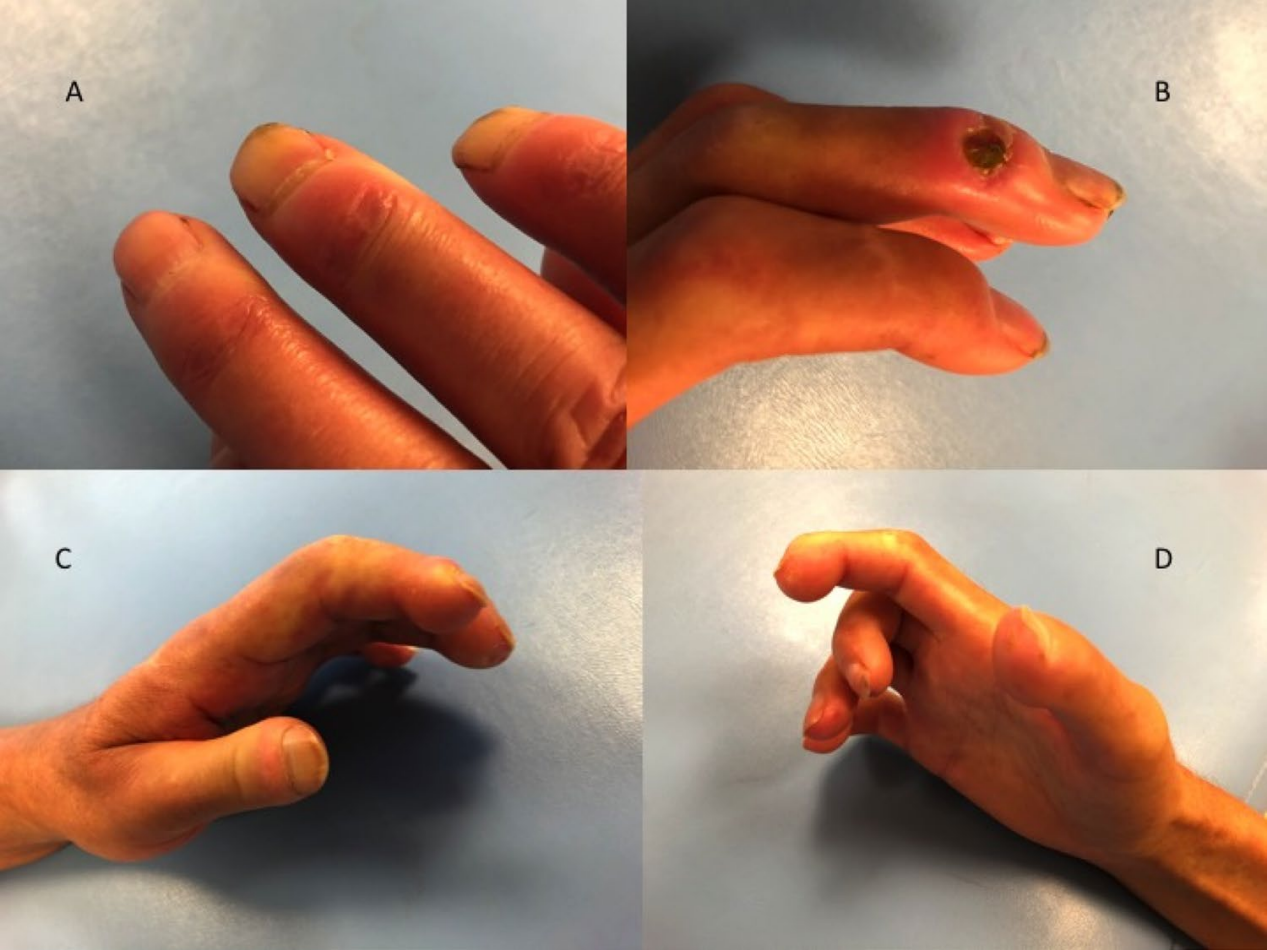
Clinical manifestations in the hands in SSc patients: thickening of the skin and subcutaneous tissue (**A**); digital ulcer (**B**); claw-type deformity of the fingers: contractures in proximal and distal interphalangeal joints (PIP, DIP) and hyperextension in metacarpophalangeal joints (MCP) (**C**,**D**).

All patients enrolled in the study underwent a clinical examination with particular attention given to the duration of the disease, presence of Reynaud’s phenomenon, trophic changes in the skin on the hands and feet, ulceration of the fingers and fingertips and previous treatment (Figs. 3, 4). The presence of accompanying pathological changes in the lungs (pulmonary fibrosis), gastrointestinal manifestations, cardiovascular involvement, renal abnormalities and hematological involvement were also noted during the examination (Table 1). The initial assessments of the participants and each follow-up assessment were performed by physicians (experienced in SSc). They were blinded to the allocation of the patients to the complex rehabilitation treatment.

### Radiological evaluations

X-ray examinations of both hands were conducted in all patients (Fig. 3). The radiological evaluation of the other joints was performed if needed.

### Rehabilitation protocol—Standardized, complex, supervised physiotherapy sessions

The same rehabilitation program was provided to all patients in the study group, and it was conducted 3 days per week for 4 weeks and lasted 1.5 h per session. The rehabilitation program consisted of the following activities: whirlpool massage with active exercises of the hand and elbow, soft tissue massage and passive manipulation of the joints. Detailed descriptions of the rehabilitation protocol can be found in the Supplementary Materials (S1). The rehabilitation sessions were performed by 1 physical therapist and 1 occupational therapist in the physiotherapy and rehabilitation outpatient department.

All patients, after completing the rehabilitation protocol, were also prescribed a daily exercise program to perform at home (lasting 30 min), consisting of flexion and extension of the fingers, abduction and adduction of the fingers in opposition of the thumb, flexion, extension, ulnar and radial deviation of the wrist, and pronation and supination of the forearm.

### Daily exercise program for the control group

The patients in the control group were instructed to perform daily home sessions (30 min) of active exercises involving flexion and extension of all the fingers in the MCP, DIP and PIP joints; opposition of the thumbs of both hands; flexion, extension, and radial and ulnar deviation of the wrists; and pronation and supination of the forearms.

### Outcomes

All SSc patients were assessed at baseline (Day 0), at the end of the 4-week rehabilitation period (Month 1), after the 3-month follow-up (Month 3), after the 6-month follow-up (Month 6) and after the one-year follow-up (Month 12) with regard to pain (VAS—visual analog scale), hand disability (DAHS—Disability of the Arm, Shoulder and Hand Questionnaire, CHFS—The Cochin Hand Function Scale), global disability (HAQ-DI—Health Assessment Questionnaire Disability Index and SHAQ—Scleroderma-HAQ), range of motion of the hand joints (d-FTP – delta finger to palm test, the Kapandji finger opposition test) and muscular strength of the hand (hand grip and pinch measurements). Detailed descriptions of the scales and measurements can be found in the Supplementary Materials (S2).

DASH was established as the primary outcome, and the VAS, CHFS, HAQ-DI, SHAQ, d-FTP – delta finger to palm test, the Kapandji finger opposition test, and hand grip and pinch measurements were established as the secondary outcomes.

### Statistical analysis

In a statistical analysis the normality of data was tested with the Shapiro–Wilk test. Due to distribution other than normal the nonparametric test were used. Comparisons of scores between groups were performed with the Manny-Whitney U test. In study group differences in scales scores in different time points were evaluated by the Friedman test with dedicated post-hoc tests. In evaluation of correlations the pooled data from all time points was used and tested with the Spearman’s rank correlation test. All calculations were performed with the Statisctica 13.1 (StatSoft, Cracow) with the p < 0.05 considered significant and with Bonferroni correction for multiple testing.

## Supplementary Information

Supplementary Information 1.

Supplementary Information 2.

## Data Availability

The datasets generated during and/or analysed during the current study are available from the corresponding author on reasonable request.
