# Supplementary material for: Role and effectiveness of complex and supervised rehabilitation on overall and hand function in systemic sclerosis patients—one-year follow-up study
Source: Sci Rep. 2021 Jul 26;11:15174. doi: 10.1038/s41598-021-94549-y (PMC8313718; doi:10.1038/s41598-021-94549-y)
Supplement: Supplementary file 1 — Supplementary Information 1. [file 41598_2021_94549_MOESM1_ESM.pdf]

The role and effectiveness of complex and supervised rehabilitation on hand and overall function in systemic sclerosis patients – one-year-follow-up.

Michał Waszczykowski [1*], Bożena Dziankowska-Bartkowiak [2], Michał Podgórski [3], Jarosław Fabiś [1], Arleta Waszczykowska [4]

Detailed descriptions of rehabilitation protocol

1. A whirlpool massage. Each patient submerged his forearm and hand in water in a whirlpool massage bath (Meden-Inmed Sp z o.o., Koszalin, Poland), maintaining a 90 degree flexion in the elbow joint. The water temperature was 40 degrees. The whirlpool massage lasted 20 minutes. During the massage, the patients performed active movements of the hand: dorsal and palmar wrist flexion, radial and ulnar deviation of the hand, pronation and supination of the foreamr and active flexion and extension of the fingers of the hand (with use of small sponge ball) and the thumb opposition. Each exercise was repeated 10 times, holding a given hand position for 10 seconds (Figure S.1).
2. A soft tissue massage. The physiotherapist performed a soft tissue massage of the forearm and hand (distal interfalangeal joints – DIP joints; proximal interphalangeal joints – PIP joints; metacarpo-phalangeal joints – MCP joints, fingers, palm, radio-ulnar joint), which lasted 20 minutes for each upper limb.
3. Passive manipulation of the joints. The physiotherapist performed passive exercises of the following joints: radio-ulnar joint (dorsal and palmar flexion, ulnar and radial deviation), MCP joints, PIP joints, DIP joints (flexion and extension of the joints) and a forearm pronation and supination. Passive joints manipulation exercises lasted 20 minutes for each upper limb (Figure S.2).

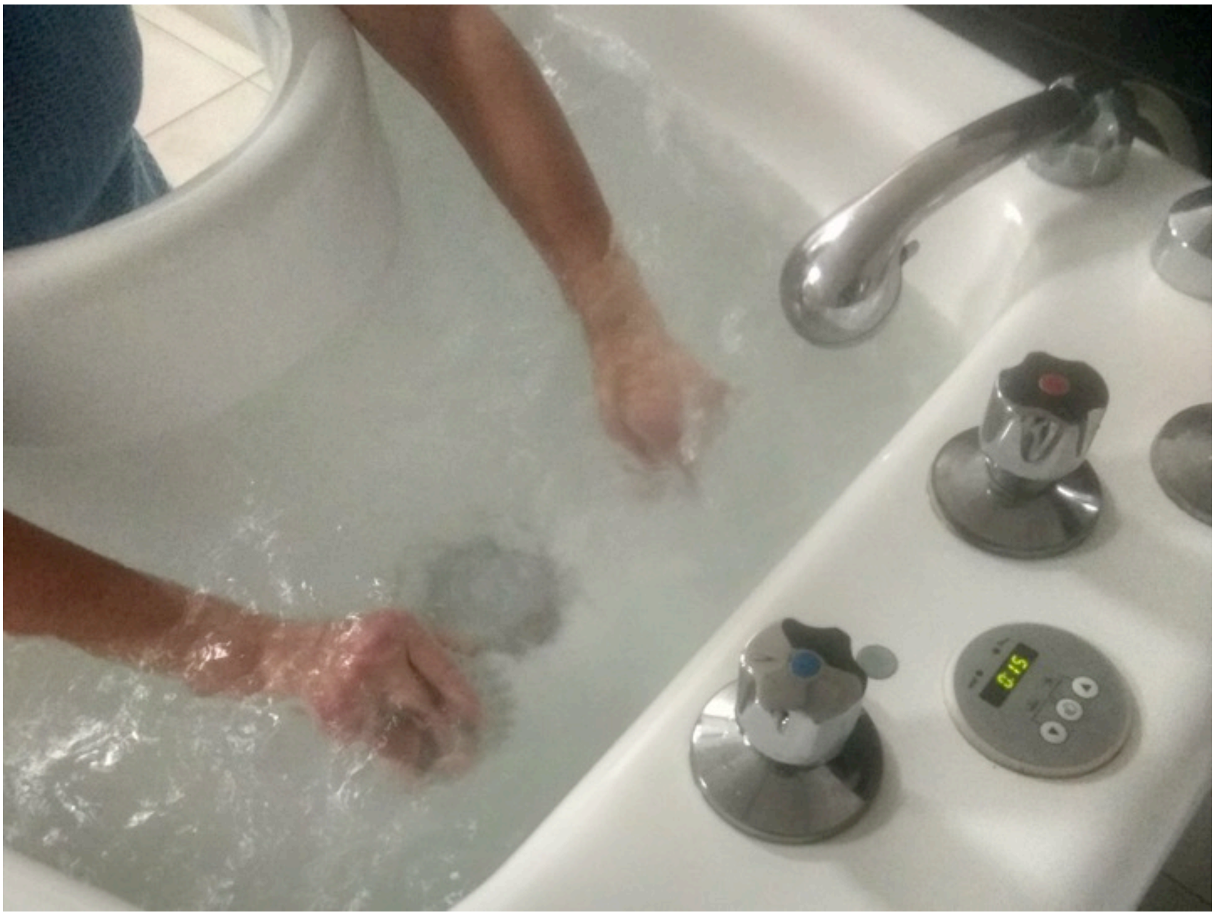

Figure S.1. Whirlpool bath with active hand and elbow exercises.

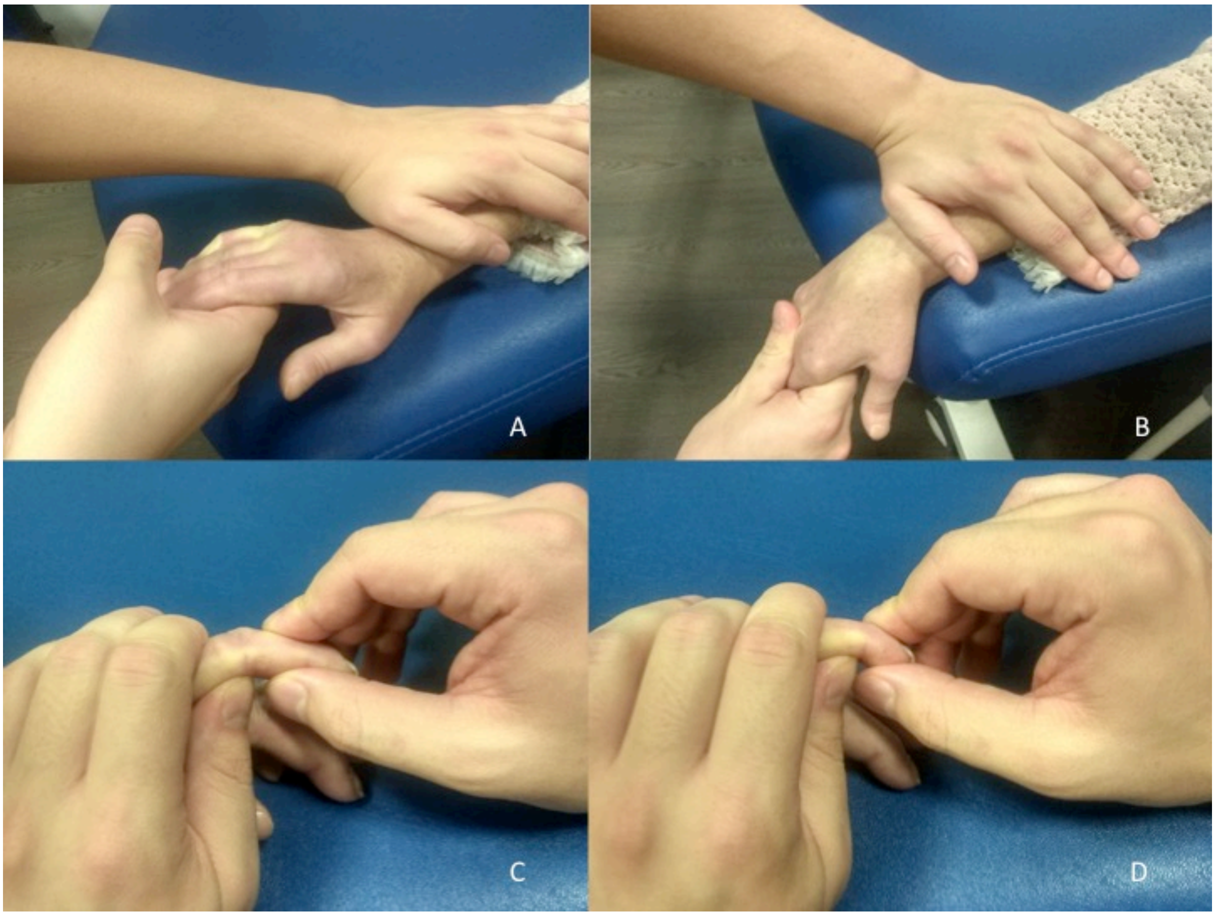

Figure S.2. Passive exercises (manipulations) of the hands' joints: wrist palmar and dorsal passive flexion (**A, B**); PIP joint passive manipulations (**C**); DIP joint passive manipulations (**D**).
