# Supplementary material for: Role and effectiveness of complex and supervised rehabilitation on overall and hand function in systemic sclerosis patients—one-year follow-up study
Source: Sci Rep. 2021 Jul 26;11:15174. doi: 10.1038/s41598-021-94549-y (PMC8313718; doi:10.1038/s41598-021-94549-y)
Supplement: Supplementary file 2 — Supplementary Information 2. [file 41598_2021_94549_MOESM2_ESM.pdf]

The role and effectiveness of complex and supervised rehabilitation on hand and overall function in systemic sclerosis patients – one-year-follow-up.

Michał Waszczykowski [1*], Bożena Dziankowska-Bartkowiak [2], Michał Podgórski [3], Jarosław Fabiś [1], Arleta Waszczykowska [4]

Detailed descriptions of scales and measurements.

Pain assessment (Visual Analog Scale – VAS)

All patients completed a questionnaire which included a visual analog scale assessing hand pain (VAS pain). The visual analog scale was a 10 cm horizontal line with "0" marked on the far left and "10" on the far right. Patients were asked to rate their level of pain of their hand from "none - 0" to "disabling - 10" by marking the scale at the pain level.

Disability of the Arm, Shoulder and Hand Questionnaire (DASH)

Disability of the Arm, Shoulder and Hand Questionnaire (DASH) is a generic questionnaire to assess upper extremity disorders. The DASH comprises of 30 questions which evaluate upper extremity functions from patients' perceptions. The DASH gives scores between 0 and 100. The higher DASH score indicates severe disability [23-25].

Cochin Hand Function Scale (CHFS)

The Cochin Hand Function Scale (CHFS) is a functional disability questionnaire comprising 18 questions about ptients daily activities: hand ability in the kitchen, during dressing, while performing personal hygiene, while performing office tasks. Each question is scored from 0 (no difficulty) to 5 (impossible to do). A total CHFS score is obtained by adding the scores from all questions (range: 0-90). Its reliability, validity, and responsiveness have been documented in rheumatoid arthritis, hand osteoarthritis, and systemic sclerosis [26, 27].

Health Assessment Questionnaire Disability Index (HAQ-DI); and Scleroderma-HAQ (SHAQ)

Health Assessment Questionnaire Disability Index (HAQ-DI) is a self-report measure, consists of twenty items grouped into eight categories of daily living: dressing and grooming, arising, eating, walking, hygiene, reach, grip, and other activitis. Persons indicate how much difficulty they have with each item from 0 (no difficulty) to 3 (cannot do). The score for each category is the highest score for any question in the category. A disability index is calculated by adding the category scores and dividing by the number

of categories answered. Index values rate from 0 (less disabled) to 3 (more disabled) [28-30].

Scleroderma-HAQ (SHAQ) consists of the HAQ (8 domains) and also includes the following VAS scales: pain, patient global assessment, vascular, digital ulcers, lung and gastrointestinal involvement. Scores on the VAS range from "0" (does not interfere with activities) to "3" (very severe limitations on activities) [31].

Finger motion (delta finger-to-palm - dFTP)

Delta finger-to-palm (d-FTP) is the difference of the distance measured between the third fingertip and the distal palmar crease with fingers in full extension and the distance with fingers in full flexion (in cm). It is a valid and reliable measure of finger motion in patients with SSc [32]. The measurements for the dominant hand were used in this study.

Kapandji score (The Kapandji finger opposition test)

The Kapandji finger opposition test was used to determine motor function of the hand. This test involves the subject attempting to touch the thumb of the hand to 10 points on the same hand in order from points 0 to 10 (Figure). The Kapandji score is the ratio of the furthest touched point on the hand to "10"[33].

Hand strength assessment (Hand grip and pinch)

To evaluate the muscular strength of the hand the grip and pinch measurements were taken. The Hydraulic Dynamometer SH5001 (SAEHAN Corporation, Changwon, 630-728, Korea) was used to measure the grip strength and Hydraulic Pinch Dynamometer SH5005 ((SAEHAN Corporation, Masan, 630-728, Korea) was used to measure tip pinch. Three measurements were taken for each grip and pinch test the mean value was calculated [34].
